# Effects of a 20-Week High-Intensity Strength Training Program on Muscle Strength Gain and Cardiac Adaptation in Untrained Men: Preliminary Results of a Prospective Longitudinal Study

**DOI:** 10.2196/47876

**Published:** 2023-10-24

**Authors:** Nicolas Pamart, Joffrey Drigny, Hélène Azambourg, Marion Remilly, Maxime Macquart, Alexandre Lefèvre, Kamal Lahjaily, Jean Jacques Parienti, Amélia Rocamora, Henri Guermont, Antoine Desvergée, Pierre Ollitrault, Francois Tournoux, Eric Saloux, Hervé Normand, Emmanuel Reboursière, Antoine Gauthier, Amir Hodzic

**Affiliations:** 1 Laboratoire Interuniversitaire de Biologie de la Motricité Université Jean Monnet Saint Etienne France; 2 Department of Sports Medicine Normandie Université Centre Hospitalier Universitaire de Caen Normandie Caen France; 3 UFR STAPS Normandie Université Caen France; 4 Department of Cardiology Normandie Université Centre Hospitalier Universitaire de Caen Normandie Caen France; 5 Centre de Recherche Clinique Centre Hospitalier Universitaire de Caen Normandie Caen France; 6 Research Center of the Montreal University Hospital Montreal University Montreal, QC Canada; 7 Inserm Comete GIP Cyceron Normandie Université Caen France

**Keywords:** actimetry, athlete’s heart, athletes, echocardiography, exercise, isokinetic dynamometry, muscle strength gain, sports physiology, strength training, strengthening exercise, untrained population, weightlifting

## Abstract

**Background:**

As strength sports gain popularity, there is a growing need to explore the impact of sustained strength training on cardiac biventricular structure and function, an area that has received less attention compared to the well-established physiological cardiac adaptation to endurance training.

**Objective:**

This study aims to implement a 20-week high-intensity strength training program to enhance maximal muscle strength and evaluate its impact on cardiac biventricular adaptation in healthy, untrained men.

**Methods:**

A total of 27 healthy and untrained young men (mean age 22.8, SD 3.2 years) participated in a strength training program designed to increase muscle strength. The training program involved concentric, eccentric, and isometric exercise phases, conducted over a consecutive 20-week time frame with a frequency of 3 weekly training sessions. Participants were evaluated before and after 12 and 20 weeks of training through body composition analysis (bioelectrical impedance), a 12-lead resting electrocardiogram, 3D transthoracic echocardiography, cardiopulmonary exercise testing, and muscle isokinetic dynamometry. The progression of strength training loads was guided by 1-repetition maximum (RM) testing during the training program.

**Results:**

Of the initial cohort, 22 participants completed the study protocol. No injuries were reported. The BMI (mean 69.8, SD 10.8 kg/m² vs mean 72, SD 11 kg/m²; *P*=.72) and the fat mass (mean 15.3%, SD 7.5% vs mean 16.5%, SD 7%; *P*=.87) remained unchanged after training. The strength training program led to significant gains in 1-RM exercise testing as early as 4 weeks into training for leg extension (mean 69.6, SD 17.7 kg vs mean 96.5, SD 31 kg; *P*<.001), leg curl (mean 43.2, SD 9.7 kg vs mean 52.8, SD 13.4 kg; *P*<.001), inclined press (mean 174.1, SD 41.1 kg vs mean 229.2, SD 50.4 kg; *P*<.001), butterfly (mean 26.3, SD 6.2 kg vs mean 32.5, SD 6.6 kg; *P*<.001), and curl biceps on desk (mean 22.9, SD 5.2 kg vs mean 29.6, SD 5.2 kg; *P*<.001). After 20 weeks, the 1-RM leg curl, bench press, pullover, butterfly, leg extension, curl biceps on desk, and inclined press showed significant mean percentage gains of +40%, +41.1%, +50.3%, +63.5%, +80.1%, +105%, and +106%, respectively (*P*<.001). Additionally, the isokinetic evaluation confirmed increases in maximal strength for the biceps (+9.2 Nm), triceps (+11.6 Nm), quadriceps (+46.8 Nm), and hamstrings (+25.3 Nm). In this paper, only the training and muscular aspects are presented; the cardiac analysis will be addressed separately.

**Conclusions:**

This study demonstrated that a short-term high-intensity strength training program was successful in achieving significant gains in muscle strength among previously untrained young men. We intend to use this protocol to gain a better understanding of the impact of high-intensity strength training on cardiac physiological remodeling, thereby providing new insights into the cardiac global response in strength athletes.

**Trial Registration:**

ClinicalTrials.gov NCT04187170; https://clinicaltrials.gov/study/NCT04187170

## Introduction

### Overview

The physiological characteristics of the athletes’ hearts in strength disciplines have gained significant attention in recent years, challenging Morganroth’s hypothesis that athletes in endurance or resistance sports have divergent cardiac remodeling phenotypes [1]. According to this postulate, endurance athletes typically exhibit eccentric left ventricular (LV) hypertrophy as a result of an increased exercise-induced cardiac preload. In contrast, the authors have reported a concentric LV hypertrophic pattern in resistance-trained athletes as an expected chronic myocardial adaptation to acute repetitive increases in LV parietal stress due to greater cardiac afterload generated by high-intensity strength training [2,3]. Experimental observations in humans have highlighted the critical role of heart-lung interaction during submaximal resistance exercise performed with a brief Valsalva maneuver, preventing an acute increase in LV systolic wall stress despite a significant augmentation in arterial blood pressure (BP) [4]. These observations have been confirmed on a larger scale, as demonstrated by the meta-analysis published by Utomi et al [5], which showed that both endurance and strength athletes present a similar pattern of LV eccentric hypertrophy. However, LV dilatation and mass increment were less pronounced in strength athletes than in endurance athletes. Nonetheless, generalizing the LV remodeling phenotype in strength athletes remains difficult, as it could be heterogeneous between diverse strength disciplines that imply various degrees of static and dynamic loads and intensity imposed during resistance training [6]. Furthermore, the potential use of anabolic androgenic steroids among strength athletes can lead to maladaptive LV concentric hypertrophy and should be screened when interpreting cardiac adaptations in strength athletes [7,8].

The right ventricular (RV) response to resistance training has not been extensively investigated, and the available data are limited. One possible reason for this is the technical challenge of accurately imaging the complex RV structure using conventional echocardiography [9], which is the preferred imaging modality for studying an athlete’s heart. Cross-sectional studies using novel techniques such as 3D echocardiography have demonstrated that RV enlargement is more prevalent in endurance athletes compared to strength athletes. Endurance training was predictive of RV dilatation, as well as the duration of the training, pulmonary artery systolic pressure, and LV stroke volume [10]. More recently, D’Ascenzi et al [11] confirmed in a large population of Olympic athletes that balanced biventricular remodeling assessed using cardiac magnetic resonance imaging seemed to be a constant characteristic of the athlete’s heart and did not appear to be influenced by the sports category. Yet, previous longitudinal studies examining the RV response to strength training programs have yielded contradictory results. Observations made among pretrained athletes with a history of chronic high-intensity resistance training using 2D echocardiography suggested that 12 weeks of American-style football competition did not involve RV morphological or functional changes, despite combined sustained endurance and resistance training loads [12]. On the other hand, American-style football interseasonal training was found to be associated with proportionate biventricular enlargement among university athletes, regardless of field position, as analyzed using 3D echocardiography [13]. Furthermore, Spence et al [14] failed to demonstrate significant cardiac morphologic changes in a small group of untrained, healthy young participants following 24 weeks of resistance training, although slight augmentations in RV mass and end-diastolic volume were observed. More recently, Scharf et al [15] demonstrated that 22 weeks of high-intensity resistance training was sufficient to induce small but significant biventricular remodeling in untrained, middle-aged men. However, the authors did not evaluate the intensity and efficiency of training through muscular testing, which may raise questions about the impact of the training program on cardiac response. Thus, data from the literature obtained from various populations, training programs, and imaging modalities do not currently allow for easy prediction of the physiological biventricular cardiac response in strength-trained athletes. Further research is necessary to improve our understanding of the RV response to resistance training and its implications for athletic performance and health.

### Aims

This study aims to investigate the cardiac biventricular adaptations to prolonged high-intensity resistance training in untrained young, healthy men.

Our three primary objectives were to (1) administer a high-intensity resistance exercise program for the upper and lower limbs that will allow for an increase in maximal muscle strength over 20 weeks of training in an untrained population; (2) objectively quantify the muscle strength gain following 12 and 20 weeks of training; and (3) analyze the changes in RV and LV volumes and functions during physical training using 2D and 3D echocardiography.

Additionally, a secondary objective was to evaluate the changes in the circadian rhythm of the sleep-wake cycle through actimetry recordings before and after the first 12 weeks of strength training.

## Methods

### Study Design

**Figure 1:**
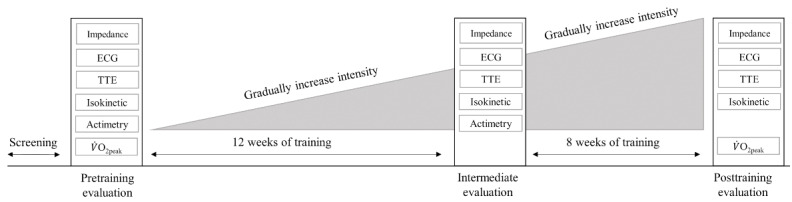
Study design. ECG: electrocardiogram; TTE: transthoracic echocardiography; VO_2peak_: maximum oxygen uptake.

We conducted a nonrandomized controlled study to analyze the effect of 20 weeks of a high-intensity strength exercise program on muscle strength gain, cardiac response, aerobic fitness, and chronobiology in a group of healthy, untrained young men. Before the beginning of the strength training program, all participants underwent clinical examination, body composition measurements, electrocardiogram (ECG), transthoracic echocardiography (TTE), and isokinetic dynamometry, which were repeated after 12 and 20 weeks of strength training. A determination of maximum oxygen uptake (VO_2peak_) by cardiopulmonary exercise testing was conducted before and after 20 weeks of strength training. Additionally, actimetry recordings were obtained to evaluate the circadian rhythm of the sleep-wake cycle before and after the initial 12 weeks of strength training. The study design is illustrated in Figure 1. Study screening and baseline evaluations were conducted on all participants within 8 weeks before the initiation of the strength training program. To minimize the effect of detraining, the 20-week evaluation was completed within 2 weeks after the end of the training program. Participants were instructed to abstain from exercise for 24 hours before data collection. TTE (with ECG and impedancemetry), VO_2peak_, and isokinetic evaluations were not carried out on the same day to avoid interference between the tests, with the exception of VO_2peak_ and isokinetic analysis for the upper limbs, which could be completed on the same day.

### Study Population

The study enrolled 27 healthy male volunteers aged between 18 and 40 years. The upper age limit was established to reduce the occurrence of unknown cardiovascular diseases among the study participants. To ensure an appropriate response to short-term training, only male participants were recruited, as they are known to exhibit a greater exercise-induced cardiac response than women [16-19]. The participants were free of known cardiovascular, liver, renal, respiratory, and metabolic disease; were not taking any prescribed medication; were nonsmokers; were not hypertensive or diabetic; and had a BMI <35 kg/m^2^. None of the participants had engaged in more than 75 minutes of sustained physical training (endurance or strength training) per week in the 3 years preceding the study. Participants presenting anomalies during the medical, ECG, or TTE examinations were excluded. None of the participants reported the use of drugs or doping products.

### Strength Training Program

The strength training program was developed in collaboration with the Departments of Sports Medicine and Sports Sciences at the University of Caen Normandy. The program involved 3 training sessions per week for a duration of 20 weeks, from January to June 2022, and was conducted in a dedicated weightlifting room equipped for the study protocol.

**Figure 2:**
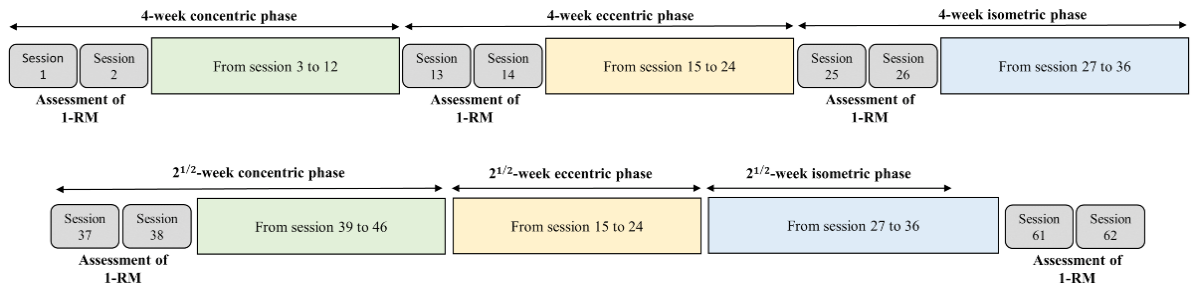
Graphic illustration of the 20-week strength training program involving cyclic concentric, eccentric, and isometric high-intensity exercises and 1-RM testing for progressive intensity adjustment. 1-RM: 1-repetition maximum.

The training sessions were supervised by sports science graduates and were limited to a maximum of 15 participants at the same time. The weightlifting room was dedicated to the protocol every evening of the week from 6 PM to 10 PM. The program used a combination of concentric, eccentric, and isometric exercise phases, intending to develop maximal strength in a short-term period. To achieve the desired strength gains, the program applied exercises performed at >70% of the participant’s 1-repetition maximum (1-RM). This type of program has previously demonstrated significant increases in muscle strength after only 8 and 12 weeks of training [20]. To monitor progress, strength exercise intensity was periodically evaluated for each exercise throughout the 20-week training period (Figure 2). Participants were required to fill out a training log to track their progress. Each training session consisted of circuit training with exercises targeting the muscle groups of the upper and lower limbs, as well as the trunk (Table 1). The strength exercises were repeated for each phase, with the sequence increasing in intensity every 2 sessions.

**Table 1:** Muscle group–specific strength training program.

Exercise	Session 1	Session 2	Session 3
Quadriceps	Leg extension	Inclined press	Leg extension
Hamstring	Leg curl	Inclined press	Leg curl
Biceps	Vertical pull	Curl biceps on desk	Vertical pull
Triceps	Bench press	Pullover	Bench press
Pectorals	Bench press	Butterfly	Bench press
Dorsal	Vertical pull	Pullover	Vertical pull

The concentric phase involved 8 repetitions (reps) at 70% of 1-RM, followed by 6 reps at 80% of 1-RM, 3 reps at 90% of 1-RM, and 2 reps at 90% of 1-RM.

The eccentric phase involved 4 sets of 6 reps at 90% of 1-RM, followed by 3 sets of 4 eccentric reps at 100% of 1-RM and 2 concentric reps at 90% of 1-RM, 3 sets of 3 eccentric reps at 110% of 1-RM and 6 concentric reps at 80% of 1-RM, 3 sets of 3 eccentric reps at 120% of 1-RM and 8 concentric reps at 70% of 1-RM, and 3 sets of 2 eccentric reps at 130% of 1-RM and 10 concentric and explosive reps at 50% of 1-RM.

The isometric phase involved 5 sets of 8 stato-dynamic reps at 50% of 1-RM, followed by 5 sets of 4 stato-dynamic reps at 50% of 1-RM and 1 rep in 20 seconds total isometric at 80% of 1-RM, 5 sets with 1 rep in 20 seconds total isometric and 4 concentric and explosive reps at 80% of 1-RM, and 5 sets with 2 reps in maximal isometric for 6 seconds and 6 concentric and explosive reps at 80% of 1-RM.

Each training session began with a 10-minute warm-up, consisting of 5 minutes of joint warm-up followed by 5 minutes of a global training circuit for the upper and lower limbs. Participants were instructed not to engage in any other physical training sessions outside of those specified in the study protocol, including recreational sports.

### Clinical Evaluation

Weight, body composition, 12-lead ECG, heart rate (HR), and arterial BP were collected at each protocol’s time points. Resting ECG (Schiller Cardiovit AT-102 G2), HR, and BP were measured after 10 minutes of quiet rest in a supine position. BP was averaged on duplicate successive measurements assessed using a Dynamap V100 automatic pressure cuff (GE Medical). BMI was calculated as follows: weight (kg)/height (m)^2^. The body surface area was calculated using the formula of Dubois [21]. The body composition was measured using an mBCA 525 impedance meter (SECA) in the supine position, as previously validated [22].

### Muscle Strength Measurement

We used a Con-Trex isokinetic dynamometer (Con-Trex MJ) for measuring upper and lower limbs muscle strength. Isokinetic testing is considered a gold standard for measuring maximal muscle strength [23], and a valid and reliable method for assessing upper and lower limbs muscle strength [24,25]. Also, isokinetic testing is appropriate for assessing the change in maximal strength after a resistance training program [26]. For both the upper and lower, the studied variables were the maximal peak torque (in Newton-meter; Nm) and the peak torques normalized to body weight (Nm/kg) assessed with a mechanical flat scale (Seca 750), which are the most reliable parameters [27]. Both the dominant and nondominant sides were tested independently [28]. A coefficient of variation of less than 10% in the maximal peak torques was mandatory to classify the effort as maximal [29].

For lower limbs, the knee flexors and extensors were tested. The testing apparatus was set up as described in the manufacturer’s owner manual, and participants were positioned in the seated position for knee flexion and extension testing, the back seat fixed at 85° of hip flexion with hands and back stabilization [30]. The lateral condyle was aligned with the dynamometer’s axis. The range of motion was set at 70°, from 90° to 20° of knee flexion, with 0° corresponding to full knee extension. All participants had a familiarization set of submaximal reps for all conditions. Data were collected from a first set of 4 maximal reps at 60°/s in the concentric mode and then a second set of 4 maximal reps at 240°/s in the concentric mode with constant verbal stimulation [31]. At all assessments, the participant’s legs were passively weighted to provide gravity compensation data, and corrections were incorporated [32]. The test-retest reliability of the Con-Trex isokinetic dynamometer for the knee joint is considered excellent, with intraclass correlation coefficients (ICCs) ranging from 0.98 to 0.99 for the measurement of maximal strength of the knee flexor and extensors [33]. Regarding the sensitivity to change of isokinetic testing, the smallest real difference for the maximal concentric strength of knee flexors and extensors are 22.75 Nm and 15.45 Nm, respectively, corresponding to 17.95% and 19.47% [25].

**Figure 3:**
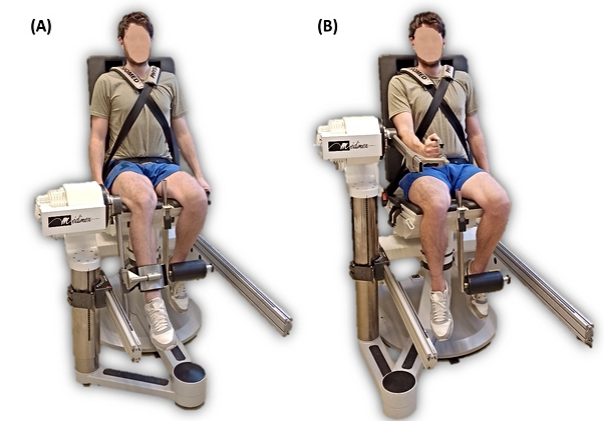
Setup for isokinetic testing of (A) the knee and (B) the elbow flexors and extensors in the seated position.

For upper limbs, the elbow flexors and extensors were tested. The testing apparatus was set up as described in the manufacturer’s owner manual, and participants were positioned in the seated position for elbow flexion and extension testing, with the forearm in the neutral position [34]. The lateral condyle was aligned with the dynamometer’s axis. The range of motion was set at 90°, from 110° to 20° of elbow flexion, with 0° corresponding to full elbow extension [35]. All participants had a familiarization set of submaximal reps for all conditions. Data were collected from a first set of 3 maximal reps at 60°/s in the concentric mode and then a second set of 4 maximal reps at 150°/s in the concentric mode with constant verbal stimulation. At all assessments, the participant’s legs were passively weighted to provide gravity compensation data, and corrections were incorporated. The test-retest reliability of isokinetic testing is considered good to excellent, with ICCs ranging from 0.83 to 0.91 for the measurement of maximal strength of elbow flexors and extensors. The ICC for the measurement of maximal strength of knee flexors and extensors ranged from 0.83 to 0.91 [36]. Figure 3 shows the setup for isokinetic testing of the knee and the elbow flexors and extensors performed in our protocol.

### Echocardiographic Analysis

The echocardiographic evaluation was conducted according to the current guidelines [37,38] using a commercially available echocardiographic system, Epiq 7 (Philips), equipped with an X5-1 xMATRIX-array transducer. The examination was performed at rest in the left lateral decubitus position using a standardized echocardiographic protocol from the parasternal (long- and short-axis) and apical (4-, 3-, and 2-chamber) views. All echocardiographic measurements acquired during a brief apnea were stored digitally for offline data analysis, to be performed blinded to the study time point using the IntelliSpace QLAB software (Philips Medical Systems). LV and RV diameters, wall thickness, and aortic sinus dimensions were assessed from B-mode acquisitions. Left and right atrial volumes were estimated using the area-length method in apical views [37]. LV and RV diastolic functions were analyzed using pulsed Doppler and tissue Doppler indices obtained as the average value of 3 consecutive cardiac cycles [38,39]. LV relaxation was evaluated from the color M-mode mitral flow propagation velocity [40,41]. Ventricular and atrial deformations were based on speckle-tracking 2D analysis. Real-time 3D full-volume acquisitions were performed for the assessment of LV and RV volumes and function, and LV mass [37].

### Cardiopulmonary Exercise Testing

All participants performed an incremental stepwise exercise test on an electronically braked ergometer (Ergoline, Ergoline GmbH), with continuous HR and 12-lead ECG monitoring. The cardiopulmonary exercise testing protocol consisted of 1 minute at rest in the sitting position, followed by a 3-minute warm-up at a load of 50 watts. The workload was increased by 30 watts every 3 minutes until exhaustion, despite verbal encouragement. BP was measured every 2 minutes using a manual BP cuff. Respiratory gas analysis was performed on a breath-by-breath basis using the Medisoft gas analyzer (ExpAir Soft) and according to previously validated methods [42]. Before and following each test, the system was calibrated according to the manufacturer’s instructions. The following parameters were determined by 2 trained and independent operators: maximum oxygen uptake (VO_2peak_), first ventilatory threshold, and second ventilatory threshold. Exercise capacity was assessed based on VO_2peak_, which was determined as the average of values obtained during the final 20 seconds of exercise. A respiratory exchange ratio greater than 1:1 and HR at peak exercise greater than 85% estimated maximal HR were used to evaluate test adequacy measures indexed to body weight.

### Actimetry Recording

For each participant, the sleep-wake cycle was recorded continuously using the Motion Watch 8 wrist-worn triaxial actigraph (CamNTech Ltd) over 7 days before and after 12 weeks of strength training. The actimeter was placed on the nondominant wrist to ensure no modification to the participant’s habits. The actigraph was worn every day and was only removed in case of prolonged immersion. The actigraphy recording was edited with the information listed in the subjective sleep diaries. The diaries were used to record the start and end (date and time) of nighttime sleep and daytime naps. The sleep diary was used to complement the actigraphy to determine when participants were awake and when they were asleep. All records were scored as awake unless (1) the sleep diary indicated that the participant was lying down attempting sleep, and (2) the activity counts from the monitor were sufficiently low to indicate that the participant was immobile. When these 2 conditions were met simultaneously, time was scored as sleep. Data recorded by the Actiwatch were downloaded and analyzed using the Motion Watch Motion Ware software (Cambridge Neurotechnology Ltd) equipped with the NPCRA (nonparametric circadian rhythm analysis) tool in 5-second epochs. The NPCRA function applies to the set of days determined by a nonlinear analysis in order to assess the activity-rest cycle. After this, the software calculates the activity-rest rhythm parameters, which are the activity during the 5 least active hours, the activity on the 10 most active hours, and the absolute amplitude of rhythm (difference between the 10 most active hours and the 5 least active hours), expressed in movement. The analysis of the rhythmicity of the activity-rest cycle is described by the amplitude of the cycle (corresponding to half of the total variability of a rhythm and half of the difference between peak and trough values, in mvts/min), by the acrophase (corresponding to the peak of the variation of this rhythm with respect to a reference phase, in hours), and by the midline-estimating statistic of rhythm (corresponding to the adjusted rhythm for a given period and the average of the values per time unit, in mvts/min). Interday stability, intraday stability, and relative amplitude were also analyzed. Parameters of interest for the sleep analysis were bedtime (hours:minutes), get-up time (hours:minutes), time in bed (TIB, hours:minutes), sleep latency (minutes), total sleep time (TST, hours:minutes), and sleep efficiency (%) defined as TST/TIB×100. Nights from Monday to Friday were considered weeknights and nights from Friday to Monday were considered weekend nights.

### Statistical Analysis

According to previously published data by our team demonstrating a significant seasonal training RV end-diastolic volume enlargement (*d*=7 mL/m^2^) among 15 American-style football competitive linemen players (being considered as strength athletes) using 3D echocardiography [13], we estimated with 80% power and a 2-tailed α of .05 that a minimum of 25 participants were required in our protocol. Participants were included in the final analysis if they performed more than 90% of the training sessions. Estimating a 10% loss of sight, 27 participants were required in this study. Statistical analyses were performed using MedCalc (MedCalc Software). The normality of the distribution was verified using the Shapiro-Wilk test. A 1-way ANOVA was used to compare time effects, and the Tukey-Kramer post hoc test was used to compare differences between each evaluation. Before the ANOVA, the Levene test for equality of variances was performed.

### Ethics Approval

The study was approved by the French research ethics committee (EUDRACT: 2019-A01235-52). Informed consent was obtained from all participants involved in the study. All the collected data were anonymous. A compensatory allowance of €150 (US $158) was provided for all participants, subject to their participation in the entirety of the study.

## Results

### Overview

A total of 22 participants completed the protocol and were included in the final analysis. Among these 22 participants, 20 completed the 60 training sessions provided by the study protocol, and 2 participants completed only 58 sessions. One participant was excluded early for medical reasons due to the discovery of unknown Arnold-Chiari syndrome, preventing him from continuing high-intensity strength training. Overall, 4 participants were excluded after the intermediate evaluation due to loss of sight. No musculoskeletal injuries were declared during the entire strength training protocol.

### Anthropometric Data

The mean age of our population was 22.8 (SD 3.2) years. The mean height and weight were 178 (SD 6) cm and 69.3 (SD 10.4) kg, respectively. The BMI was in a normal range and remained stable. Only 2 participants had a BMI between 25 kg/m^2^ and 30 kg/m^2^. None of the participants presented a BMI <18 kg/m^2^. As shown in Table 2, the body composition variables (body fat and lean body mass) remained unchanged over the following period.

**Table 2:** Anthropometric data.

Variable	Pretraining, mean (SD)	12-week training, mean (SD)	20-week training, mean (SD)	Interaction, *P* value
Weight (kg)	69.8 (10.8)	72.1 (10.1)	72 (11)	.72
BMI (kg/m^2^)	22.1 (3)	22.8 (2.7)	22.8 (3)	.66
Body fat mass (kg)	11.3 (6.8)	11.7 (7)	12.4 (6.8)	.87
Body fat mass (%)	15.3 (7.5)	15.6 (7.8)	16.5 (7)	.87
Lean body mass (kg)	58.5 (6.1)	60.4 (7)	59.6 (6.5)	.63
Lean body mass (%)	84.7 (7.5)	84.4 (7.8)	83.5 (7)	.87

### Muscular Effects

The muscle gain assessment using isokinetic testing is shown in Table 3. For the lower limbs, significant improvements in muscle strength were observed as early as 12 weeks of strength training for both the quadriceps (mean ∆_0-12wks_ were +20.5% and +20.2% for the right and left quadriceps, respectively) and the hamstrings (mean ∆_0-12wks_ were +16.6% and +18.8% for the right and left hamstrings, respectively) compared to the pretraining evaluation. After 20 weeks of training, the mean percentage of muscular gain was close to +28% for all tested muscles of the lower limbs. Between 12 and 20 weeks of training, the muscle strength tended to increase without reaching significance (mean ∆_12-20wks_ for the quadriceps was +7.25% and mean ∆_12-20wks_ for the hamstrings was +9.05%). For the biceps and triceps strength, no significant differences were found after 12 weeks of training (Table 3). However, at the end of the 20-week training program, the mean strength of the triceps increased significantly by +21.6% for the right side and +24.3% for the left side. Concerning the biceps strength, the mean strength was significantly improved by +18.2% for the right side. The left biceps strength increased by +16.9% (*P*=.10).

The kinetics of the 1-RM evaluations, presented in Table 4, confirmed the efficiency of the strength-training program with highly significant improvements after 20 weeks compared to baseline (>40% for the 1-RM leg curl, >41.1% for the 1-RM bench press, >50.3% for the 1-RM pullover, >63.5% for the 1-RM butterfly, >80.1% for the 1-RM leg extension, >105% for the 1-RM curl biceps on desk, and >106% for the 1-RM inclined press). Significant differences were observed as early as 4 weeks of training for the 1-RM testing, except for the 1-RM bench press and pullover which were significantly improved after 8 weeks of training (Table 4).

Please take note that the evaluation of the strength training program’s effects on cardiac function, aerobic fitness, and actimetry recordings will be analyzed and presented separately.

**Table 3:** Muscle strength gain assessment using isokinetic dynamometry.

Variable	Pretraining, mean (SD)	12-week training, mean (SD)	20-week training, mean (SD)	Interaction, *P* value
Right biceps (Nm)	51.1 (12.4)	59.1 (13.1)	60.4 (9.7)^a^	.02
Left biceps (Nm)	53.2 (10.8)	57.3 (14.3)	62.2 (15.6)	.10
Right triceps (Nm)	50 (12.1)	57.5 (12.4)	60.8 (9.2)^a^	.008
Left triceps (Nm)	51.5 (10.9)	59.9 (12.3)	64 (12.7)^a^	.003
Right quadriceps (Nm)	160.3 (34)	193.1 (26.9)^a^	207.3 (42.3)^a^	<.001
Left quadriceps (Nm)	162.2 (35.9)	194.9 (38.1)^a^	208.8 (45.1)^a^	.001
Right hamstring (Nm)	90 (21.4)	104.9 (22.7)^a^	115.5 (28.2)^a^	.004
Left hamstring (Nm)	88.6 (20.1)	105.3 (22.1)^a^	113.7 (24.3)^a^	.001

^a^*P*<.05 significantly different from pretraining (ANOVA).

**Table 4:** Changes in the 1-repetition maximum (1-RM) assessment during the strength training program.

Variable	Pretraining, mean (SD)	4-week training, mean (SD)	8-week training, mean (SD)	12-week training, mean (SD)	20-week training, mean (SD)	Interaction, *P* value
1-RM leg extension (kg)	69.6 (17.7)^a^	96.5 (31.9)^b^	102.9 (21.6)^b^	110.9 (22.4)^b^	125.4 (24.6)^b^	<.001
1-RM leg curl (kg)	43.2 (9.7)^a^	52.8 (13.4)^b^	54.2 (10.5)^b^	56.8 (10.6)^b^	60.5 (12)^b^	<.001
1-RM bench press (kg)	54.8 (11.8)^a^	61.5 (12.5)	68.2 (13.5)^b^	72.4 (13.2)^b^	77.3 (14.2)^b^	<.001
1-RM inclined press (kg)	174.1 (41.1)^a^	229.2 (50.4)^a,b^	278.8 (62.1)^b^	304 (63.8)^b^	358 (92.7)^b^	<.001
1-RM butterfly (kg)	26.3 (6.2)^a^	32.5 (6.6)^a,b^	37.8 (6.9)^b^	39.7 (6.9)^b^	43 (7.8)^b^	<.001
1-RM curl biceps on desk (kg)	22.9 (5.2)^a^	29.6 (5.2)^a,b^	35.9 (6.9)^b^	39.8 (7.2)^b^	47 (7.9)^a,b^	<.001
1-RM pullover (kg)	38 (9.7)^a^	43.8 (8.9)	47.7 (8.4)^b^	51.7 (9.4)^b^	57.1 (15)^b^	<.001

^a^*P*<.05 different from the 1-repetition maximum at 12-week training (ANOVA).

^b^*P*<.05 different from the 1-repetition maximum at pretraining.

## Discussion

### Overview

This study protocol was designed to evaluate the physiological effects of a prolonged, high-intensity 20-week resistance training program on untrained male participants. This study aimed to describe the relationship between muscular and cardiac responses using isokinetic measures of muscle strength gain and a 3D echocardiographic assessment of biventricular cardiac adaptation. The novelty of our approach was to establish the validity of an innovative strength-training program meeting the conditions of duration, intensity, and tolerance, which allowed us to demonstrate its effectiveness in increasing muscle strength in both lower and upper limbs. This is a prerequisite for interpreting cardiovascular adaptation to strength training.

Regular resistance training, either coupled with an endurance exercise program or not, has become an important factor in promoting public health due to its proven beneficial effects on the improvement and maintenance of physical fitness and cardiovascular health in adults with or without chronic diseases or disabilities [43-46]. On the other hand, the increasing popularity of sustained heavy weightlifting training programs has prompted the scientific community to better understand cardiovascular acute and chronic responses to heavy strength exercise, which is responsible for the extreme but transitory elevation of cardiac afterloads, and to analyze the cardiac phenotype observed in trained weightlifters [1,47]. However, despite a few longitudinal observations studying the relationship between resistance training and chronic cardiac adaptation in healthy, untrained participants using cardiac imaging, some methodological limitations in terms of assessing muscle strength gain and controlling the intensity of the strength training program may have limited the interpretation of exercise-induced cardiac response [14,15,48]. The majority of these studies have only assessed muscle strength gain using the 1-RM technique. Although 1-RM is a reproducible measurement, it may overestimate muscle strength gain, particularly for higher strength levels, when compared to the gold-standard isokinetic evaluation [49]. Therefore, it could bias the interpretation of the effectiveness of a strength-training program and its impact on cardiovascular properties. The findings of this study are in line with previous research. Our results of the 1-RM technique revealed that the average increase in muscle strength was at least 40% for the leg curl and up to 106% for the inclined press, despite only a 30% gain being observed with isokinetic evaluation.

To achieve rapid muscle strength gains over the study period, we developed a specialized strength training program for our untrained participants. The program involved alternating between concentric, eccentric, and isometric resistance exercise phases performed at high loads and maintaining a constant volume of 3 training sessions per week without any rest periods throughout the program. This training mode and intensity have previously been shown to be effective in stimulating muscle strength gains after only 12 weeks of training [20,50-52]. Our results confirmed that the maximum muscle strength of the quadriceps and hamstrings significantly increased among untrained individuals after 12 weeks of training, with lesser gains observed between weeks 12 and 20. This result was expected as our protocol was biphasic with a shorter second training period, and strength training among untrained individuals leads to rapid initial strength gains followed by a slowed progression as the muscle adapts [53,54]. In contrast, concerning the 1-RM technique, the majority of 1-RM loads significantly increased after only 4 weeks of training, except for the bench press and pullover tests, which reached significance after 8 weeks of training. Early increases in muscle strength after only 2 to 4 weeks of heavy strength training were previously linked to neuromuscular and connective tissue adaptations [53,55]. The 1-RM evaluations progressively increased throughout the study training protocol, which was supervised to allow for continuous and individualized adaptation of loads to maintain high-intensity training. The absence of a recovery period during the protocol did not seem to affect the safety of our high-intensity strength training program, as no musculoskeletal injuries were reported and over 80% (22/27) of participants completed the training with an average of 59.8 training sessions out of the 60 sessions planned in the study protocol. In comparison, longitudinal research by Spence et al [14,56] analyzing cardiac adaptation in a small group of untrained young men undergoing either resistance or endurance training for 24 weeks showed a small trend toward biventricular eccentric remodeling in strength athletes. The authors reported only a 19.2% increase in the 1-RM bench press and 55% in the squat exercise. As pointed out by the authors, the intensity of the strength-training program was questionable as it involved a first low-intensity 12-week preparatory phase with exercise performed at low volume and load and interspersed with weeks of recovery [14,56]. This raises the question of whether higher-intensity strength training would have resulted in greater cardiac physiological adaptation in the resistance-trained participants. Moreover, to ensure that participants did not perform unseen sustained endurance exercises during this study that could affect the cardiac response, measurements of maximal oxygen consumption were performed before and after the training program. Specific strength training does not significantly improve aerobic fitness, unlike endurance exercise [57].

### Conclusion

This study protocol has provided evidence supporting the efficacy of our weightlifting training program in increasing maximal muscle strength among untrained healthy young males over 20 weeks. This will enable us to further explore the physiological relationship between exercise-induced cardiac response and muscle strength gain in weightlifters through a comprehensive cardiovascular evaluation using ECG, TTE, and CPET. Moreover, the findings of this study have the potential to contribute to the development of research protocols aimed at deepening our understanding of the physiology of strength athletes.

## Abbreviations

1-RM: 1-repetition maximum
BP: blood pressure
ECG: electrocardiogram
HR: heart rate
ICC: intraclass correlation coefficient
LV: left ventricle
NPCRA: nonparametric circadian rhythm analysis
reps: repetitions
RV: right ventricle
TIB: time in bed
TST: total sleep time
TTE: transthoracic echocardiography
VO_2peak_: maximum oxygen uptake

## Data Availability

The data sets generated during the study protocol are available from the corresponding author on reasonable request.
